# High dietary total antioxidant capacity is associated with a reduced risk of hypertension in French women

**DOI:** 10.1186/s12937-019-0456-0

**Published:** 2019-06-11

**Authors:** Paola Villaverde, Martin Lajous, Conor-James MacDonald, Guy Fagherazzi, Fabrice Bonnet, Marie-Christine Boutron-Ruault

**Affiliations:** 1 Center for Research on Population Health, INSP (Instituto Nacional de Salud Pública), Cuernavaca, México; 2 INSERM (Institut National de la Santé et de la Recherche Médicale) U1018, Center for Research in Epidemiology and Population Health (CESP), Institut Gustave Roussy, Villejuif, France; 3 Université Paris-Saclay, Université Paris-Sud, Villejuif, France; 4 Department of Global Health and Population, Harvard T.H. Chan School of Public Health, Boston, MA USA; 5 Université Rennes1, F-35043 Rennes, France; 6 CHU Rennes, F-35033 Rennes, France

**Keywords:** Dietary total antioxidant capacity, Hypertension, TRAP assay, Women

## Abstract

**Background:**

Although there is evidence for a reduced risk of hypertension associated with fruit and vegetable consumption, the relationship between the total antioxidant capacity of the diet (TAC) and the risk of hypertension has not been previously examined. We aimed to evaluate that association in the large E3N French prospective cohort of women.

**Methods:**

Dietary TAC was estimated using total radical-trapping ability parameter (TRAP) assay food values; self-reported incident hypertension cases were validated. Cox regression models were adjusted for conventional risk factors, body mass index, physical activity, energy, sodium, magnesium, omega-3 fatty acids, and alcohol.

**Results:**

After an average 12.7 years of follow up, there were 9350 incident cases of hypertension among 40,576 women. Dietary TAC was inversely associated with the risk of hypertension with a 15% lower risk of hypertension in those in the fifth vs. first quintile (HR_Q5_ 0.85 [CI 95% 0.74; 0.95] p-trend 0.03) An inverse dose-effect relationship was observed for dietary TAC excluding coffee (HR_Q5_ 0.85 [CI 95% 0.74; 0.95], p-trend 0.0008), while for dietary TAC from coffee, only the highest quintile was inversely associated with risk (HR_Q5_ 0.86 [0.75, 0.97], p-trend 0.20). In a fully partitioned model with major dietary TAC contributors, TAC from fruit/vegetables, wine, and miscellaneous sources was inversely associated with risk, while associations with TAC from coffee, tea, and chocolate were not statistically significant.

**Conclusions:**

In a large prospective cohort, the risk of incident hypertension in women was inversely associated with the antioxidant capacity of the diet, suggesting that promoting a diet naturally rich in antioxidants might help prevent the development of hypertension.

**Electronic supplementary material:**

The online version of this article (10.1186/s12937-019-0456-0) contains supplementary material, which is available to authorized users.

## Background

Oxidative stress, a state of imbalance between pro-oxidants and antioxidants leading to potential alterations in endothelial cells, has been suggested to be a potentially important mechanism in the development of hypertension [1]. Antioxidants are agents that inhibit oxidation and neutralize the negative effects of free radicals. Research on antioxidants initially considered specific antioxidant vitamins such as vitamins C and E, and pro-vitamins like beta-carotene, leading to intervention studies with high dosages of those specific vitamins to prevent diseases, including cancer or cardiovascular disease. However, this approach has proven inefficient or even deleterious with increased rather than decreased risks of cancer or cardiovascular events and mortality [2–4]. In parallel, research has identified the important antioxidant potential of other components from foods of vegetable origin, especially polyphenols, in relation to risk of hypertension [5, 6]. This led to devising in vitro assays to assess the total antioxidant capacity (TAC) of different foods or whole diets instead of quantifying individual dietary antioxidant nutrient intakes. To do this, methods have been developed mostly based on hydrogen atom transfer (HAT) reactions and single electron transfer (SET) [7]. HAT based assays measure the ability of antioxidants to scavenge free radicals, thus interrupting the oxidizing chain reactions. The total radical-trapping ability parameter (TRAP) is a HAT assay for measuring dietary TAC [8]; it is a largely used method shown to correlate well with other HAT-based assays.

Intake of antioxidants has been correlated with higher antioxidant capacity of the plasma mostly in short-term trials, and results are not consistent across studies [9–15]. Indeed, plasma TAC concentrations result from the balance between pro- and anti-oxidants, and can also vary according to physiological state and genetic polymorphisms that regulate absorption, and are only a snapshot of circulating TAC. Considering the mean daily TAC intake could provide a view of the ability of the diet to reduce the risk of specific conditions, and would be effective in terms of prevention advice. Dietary TAC has been inversely associated with gastric and colorectal cancers [16, 17], myocardial infarction [18], stroke [19], and type 2 diabetes risks [20], and with total mortality [21]. To our knowledge, no study examined the association between dietary TAC and the incidence of hypertension. Therefore, we estimated dietary TAC from an available database [22, 23] and considered it in relation with risk of incident hypertension in a large cohort of middle aged women.

## Methods

### Study population

The Etude Epidémiologique de femmes de la Mutuelle Générale de l’Education (E3N) is a French prospective cohort started in 1990 comprising 98,995 women aged 40–65 years at baseline and insured by the MGEN (Mutuelle Générale de l’Education Nationale). The objective of E3N was to study the main risk factors of cancer and chronic diseases. The E3N is the French component of the European Prospective Investigation into Cancer and Nutrition [24]. The cohort received ethical approval from the French National Commission for Computerized Data and Individual Freedom (Commission Nationale Informatique et Libertés), and all participants in the study signed an informed consent.

Participants returned mailed questionnaires on lifestyle information and disease occurrence [25]. Questionnaires were completed every 2 to 3 years. The average response rate at each questionnaire cycle was 83%, and the total loss to follow-up was 3%.

In 1993, 74,520 participants responded to a follow-up questionnaire that included a validated self-administered diet history questionnaire [26]. We excluded women with an unrealistic energy consumption defined as extreme values for the ratio between energy intake and required energy (the 1st and 99th percentiles of the distribution in the population, *n* = 1381), with no follow-up after 1993 (*n* = 848), with no information on risk factors prior to 1993 (*n* = 2793), and women who reported a prevalent cancer (*n* = 4253), hypertension (*n* = 24,222), or cardiovascular disease (*n* = 440) before or at the 1993 questionnaire. The final study population included 40,576 women.

### Dietary data and dietary antioxidant capacity assessment

In 1993, dietary data was collected using a two-part 208-item self-administered diet history questionnaire. The validity and reproducibility of the dietary questionnaire has been evaluated [26]. The first part assessed consumption frequencies and portion sizes of sixty-six food groups and items. Frequency was quantified in eleven potential categories: never or less than once a month; 1, 2, or 3 times a month, and 1 to 7 times a week. The questionnaire was sent with a photo booklet to facilitate the estimation of portion sizes [27]. In a second part, qualitative questions enabled us to disentangle the considered food groups into consumption of 208 food items or beverages.

The dietary total antioxidant capacity (TAC) has already been investigated in two previous studies based on the E3N cohort [20, 21]. Dietary TAC was estimated using an Italian database [22, 23]. The TRAP (Total Radical-trapping Antioxidant Parameter) assay estimated the TAC of foods based on the transfer of hydrogen to stabilize a free radical [28]. For each food item in the diet history questionnaire, we identified an equivalent food in the TAC database. For four items (apple, melon, beer, and vinegar), more than two values were available and we calculated an average of the available values. When we failed to find a direct match, we used values for a similar food, based on the similarity in botanical group and vitamins C and E, and polyphenol contents. TRAP from coffee represented 75% of overall dietary TRAP, 6% for fruit, 5% for wine, 5% for tea, 4% for vegetables, 3% for chocolate, and 3% for other sources. Because of the dominant participation of coffee, and because of doubts about the proportion of polyphenols from coffee to be absorbed and play an actual systemic role, we decided to also present models with a partition of TRAP into coffee-TRAP and non-coffee TRAP, with mutual adjustment. For non-coffee TRAP, major providers were fruit (22%), wine (20%), tea (18%), vegetables (16%), chocolate (12%), and other miscellaneous sources (12%).

Antioxidant supplement intake was assessed through the 1995, 2000, 2002, and 2005 questionnaires. Participants were asked about their intakes of different vitamins and minerals, including vitamin E, vitamin C, and beta-carotene if consumed at least three times per week.

We estimated the energy and nutrient intakes by multiplying the quantity of daily consumption of each food by their nutrient content provided by a food composition table adapted from the French food composition table [29].

### Hypertension assessment

Participants were asked to report whether they had hypertension at baseline (1993) and in each follow-up questionnaire (1994, 1997, 2000, 2002, 2005, and 2008), the date of diagnosis, and the use of antihypertensive treatments. The month and year of diagnosis were provided for most cases (69%). For individuals who were missing the month of diagnosis (14% of cases), it was imputed to June of the year of diagnosis. The median time between the date of diagnosis and the date of response to the first questionnaire after diagnosis was 12 months. Thus, for the cases (*n* = 17%) with no year of diagnosis we assigned it to be 12 months before they reported hypertension in a questionnaire. In 2004, a drug reimbursement database became available for 97.6% of participants. We used the self-reported date of diagnosis or the first date of drug reimbursement for antihypertensive medications (Anatomical Therapeutic Chemical Classification System codes C02, C03, C07, C08, and C09) whatever happened first, as the date of diagnosis for cases identified after 2004.

In addition, using the information of the MGEN health insurance plan drug claim database, we assessed the validity of self-reported hypertension within the E3N cohort. We compared hypertension self-report to antihypertensive drug reimbursement (any of the above specified codes). A positive predictive value of 82% was observed among women alive in January 2004 and followed up to their response to the last considered questionnaire in 2008.

### Assessment of covariates

We used information from the 1992 questionnaire whenever possible. Treated hypercholesterolemia, family history of hypertension, and smoking were based on self-reports, and for diabetes we used validated cases [30]. We assessed usual physical activity with a questionnaire in 1993 [31] that included questions on weekly hours spent walking, cycling, and performing light and heavy household chores, or recreational activities (e.g., swimming and tennis), and on the daily number of climbed flights of stairs. Metabolic equivalents (MET) per week were estimated by multiplying the yearly average METs for each item based on values from the Compendium of Physical Activities [32] by the reported activity duration.

Self-reported height and weight were used to calculate body mass index (BMI), defined as weight (kg) divided by squared height (m^2^). In the cohort, self-reported anthropometry has proven reliable in a validation study [33].

Mean daily intakes of energy (excluding energy from alcohol), alcohol, magnesium, potassium, omega-3 fatty acids, and total antioxidant capacity were estimated from the dietary questionnaire in 1993 as described above.

### Statistical analysis

We categorized participants according to the TAC intake in quintiles with the lowest category as the reference. Time at entry was the age at the beginning of follow-up (1993), exit time was the age when participants were diagnosed with hypertension, died (dates of death were obtained from the participants’ medical insurance records), were lost to follow-up, or were censored at the end of the follow-up period (June 25, 2008), whichever occurred first. Hazards ratios and 95% confidence intervals were estimated from Cox regression models with age as the time scale. We estimated linear trend across categories with a semi-quantitative variable based on the median values of exposure categories.

Multivariable models were first adjusted for energy (Model 1), then for family history of hypertension (yes/no), BMI (< 25, 25–29.9.9, ≥30), physical activity (Met-h/week, continuous), smoking (time-dependent, as never, former, and current), education (no high school diploma, high school diploma), diabetes (yes/no), and hypercholesterolemia (yes/no) (Model 2); Model 3 was further adjusted for intakes of alcohol, coffee, magnesium, potassium, omega-3 fatty acids (all as continuous variables). When data for covariates were missing for less than 5% of the participants, we replaced the missing values with the median values (continuous variables) or the modal value (categorical variables).

TAC was modeled globally in quintiles, then in a partition model considering TAC from coffee (representing 75% of overall TAC) and TAC from other sources (non-coffee TAC), in a partition model, then finally in a fully partitioned model, simultaneously considering coffee TAC, TAC from fruit and vegetables, TAC from wine, TAC from chocolate, TAC from tea, and TAC from miscellaneous other sources. We designed spline regression curves to better characterize the shape of the association between non-coffee TAC and hypertension. We chose the minimum TRAP value as the reference for estimating HRs and 95% CI, and used four knots (the 20th, 40th, 60th, and 80th percentiles of the distribution).

To investigate the potential effect modification of BMI, tobacco smoking (ever/never), or energy on the association between dietary TAC consumption and incidence of hypertension, we tested the statistical significance of an interaction term between TAC consumption and the potential effect modifiers. For BMI we used 25 as the threshold value, and the median value for energy intake.

We performed sensitivity analyses to test for potential reverse causation; models excluded cases that occurred in the five first years of follow-up. We also performed the same analyses excluding women with any antioxidant supplement intake (vitamin E, vitamin C, or beta-carotene). All statistical analyses used SAS 9.3 (SAS Institute Inc., Cary, NC).

## Results

After an average 12.7 years of follow up and 493,895 person-years, we identified 9350 cases of incident hypertension (18.9 cases per 1000 person-years). The mean age at baseline was 51.6 ± 6.2 years, and the mean dietary total antioxidant capacity (TAC) consumption was 20.3 ± 14.1 mmol/day; it was 5.2 ± 1.7 mmol/day in the first quintile, and 42.1 ± 12.7 mmol/day in the fifth quintile. Baseline characteristics of participants according to quintiles of dietary TAC are listed in Table 1.

**Table 1:** Population characteristics according to dietary total antioxidant capacity (TAC) E3N Cohort, France 1993–2008

	Dietary total antioxidant capacity (TAC) (mmol/day)				
Characteristics	Q1(< 8.2)	Q2 (8.2–14.4)	Q3 (14.4–21.1)	Q4 (21.1–29.9)	Q5 (> 29.9)
Dietary TAC Intake: mmol/d (SD)	5.2 (1.7)	11.3 (1.8)	17.7 (1.9)	25.1 (2.5)	42.1 (12.7)
Risk factors					
Age at 2003: years (SD)	52.0 (6.2)	52.3 (6.5)	51.7 (6.2)	51.4 (6.1)	50.7 (5.6)
Body mass index: kg / m^2^ (SD)	21.8 (2.6)	22.1 (2.6)	22.2 (2.7)	22.4 (2.7)	22.5 (2.8)
Diabetes n (%)	46 (0.57)	36 (0.44)	38 (0.47)	34 (0.42)	38 (0.47)
Hypercholesterolemia: n (%)	397 (4.89)	362 (4.46)	404 (4.98)	402 (4.95)	339 (4.18)
Smoking: n (%)					
Never	5338 (65.78)	4523 (55.74)	4200 (51.75)	3806 (46.90)	3282 (40.44)
Former	2255 (27.79)	2744 (33.81)	2827 (35.83)	2960 (36.48)	2945 (36.29)
Current	522 (6.43)	848 (10.45)	1089 (13.42)	1349 (16.62)	1888 (23.27)
Education: n (%)					
With high school diploma: n (%)	7239 (89.21)	7369 (90.81)	7301 (89.96)	7350 (90.57)	7356 (90.65)
Family history of hypertension: n (%)	2397 (29.54)	2258 (27.83)	2348 (28.93)	2273 (28.01)	2364 (29.13)
Physical activity: METS/week (SD)	53.7 (29.2)	53.7 (29.5)	53.8 (29.4)	55.0 (30.0)	54.8 (30.7)
Dietary factors mean (SD)					
Energy without alcohol (kcal / d)	2050.6 (516.6)	2105.7 (514.6)	2116.6 (523.8)	2148.0 (543.0)	2216.0 (577.0)
Alcohol (g / day)	6.1 (8.1)	10.8 (12.3)	12.4 (13.4)	13.8 (15.0)	14.4 (16.0)
Coffee intake (ml / day)	16.8 (28.2)	111.3 (60.9)	242.1 (61.5)	384.8 (65.6)	708.6 (249.2)
Potassium (mg / day)	3637.6 (1043.2)	3760.0 (1006.1)	3759.6 (969.7)	3859.1 (941.7)	4229.3 (1072.0)
Magnesium (mg / day)	317.6 (84.1)	365.1 (81.0)	415.1 (76.7)	479.7 (77.0)	625.5 (136.2)
Omega-3 fatty acids (mg / day)	1.41 (0.50)	1.48 (0.52)	1.51 (0.54)	1.55 (0.54)	1.61 (0.57)
Sodium (mg / day)	2749.2 (893.6)	2796.1 (878.2)	2805.9 (871.9)	2847.8 (891.6)	2924.5 (942.6)
Western diet	- 0.3 (0.8)	- 0.2 (0.8)	0.0 (0.9)	0.1 (0.9)	0.3 (1.1)
Healthy diet	- 0.1 (0.9)	0.0 (1.0)	0.1 (0.9)	0.0 (1.0)	0.1 (1.0)

TAC intake was inversely associated with hypertension in the fully adjusted model (Table 2, M3) (HR_5vs1quintile_: 0.85, 95% CI: 0.74, 0.95, p-trend = 0.02). When we partitioned TAC into coffee and non-coffee TAC (Table 3), non-coffee TAC was associated with reduced risk of hypertension from the second quintile on, with an inverse dose-effect relationship (M3: HR_5vs1quintile_: 0.85, 95% CI: 0.79, 0.92, p-trend< 0.0001). Regarding coffee TAC, the association was weaker, with an inverse association only with the fifth quintile (M3: HR_5vs1quintile_: 0.86, 95% CI: 0.75, 0.97, p-trend 0.03). We further evaluated associations between dietary TAC from major TAC providers and risk of hypertension, using a fully partitioned model. Only dietary TAC from wine, from fruit and vegetables, and from miscellaneous other sources remained inversely associated with the risk of hypertension, while TAC from coffee, tea, or chocolate was not (Table 4).

**Table 2:** Hazard ratios of hypertension according to dietary total antioxidant capacity intake including coffee. E3N Cohort, France 1993–2008 (*N* = 40,576)

Dietary TAC (mmol/day)		M1		M2		M3	
	N (%) cases	HR [95% CI]	p-trend	HR [95% CI]	p-trend	HR [95% CI]	p-trend
Q1 (< 8.2)	1832 (19.6)	Reference	0.008	Reference	0.21	Reference	0.02
Q2 (8.2–14.4)	1864 (19.9)	1.01 [0.94; 1.07]		1.00 [0.94; 1.07]		0.98 [0.91; 1.04]	
Q3 (14.4–21.1)	1893 (20.2)	1.05 [0.99; 1.12]		1.02 [0.96; 1.09]		0.96 [0.89; 1.03]	
Q4 (21.1–29.9)	1920 (20.5)	1.09 [1.02; 1.16]		1.05 [0.99; 1.12]		0.95 [0.87; 1.04]	
Q5 (> 29.9)	1841 (19.7)	1.08 [1.01; 1.15]		1.01 [0.94; 1.08]		0.85 [0.74; 0.95]	

M1: Age as the time scale + energy without alcohol

M2: M1 + (Diabetes, treated hypercholesterolemia, education, family history of hypertension, smoking, physical activity, body mass index)

M3: M2 + Na (mg), K (mg), Mg (mg), AGPIw3 (mg), alcohol (g)

**Table 3:** Mutually adjusted analysis. Hazard ratios of hypertension according to dietary total antioxidant capacity intake in women, partitioned into TAC from coffee and TAC from all other sources. (*N* = 40,576). E3N Cohort, France 1993–2008

Dietary TAC (mmol/day)		M1		M2		M3	
	N (%) cases	HR [95% CI]	p-trend	HR [95% CI]	p-trend	HR [95% CI]	p-trend
non-coffee TAC							
Q1 (< 2.95)	1948 (20.8)	Reference	0.001	Reference	0.006	Reference	< 0.0001
Q2 (2.95–3.99)	1909 (20.4)	0.95 [0.89; 1.01]		0.94 [0.88; 1.00]		0.93 [0.87; 0.99]	
Q3 (3.99–5.52)	1831 (19.6)	0.89 [0.84; 0.96]		0.90 [0.84; 0.96]		0.88 [0.82; 0.94]	
Q4 (5.52–6.69)	1845 (19.7)	0.91 [0.85; 0.97]		0.91 [0.85; 0.97]		0.88 [0.82; 0.94]	
Q5 (> 6.96)	1817 (19.4)	0.90 [0.90; 0.83]		0.90 [0.84; 0.97]		0.85 [0.79; 0.92]	
Coffee TAC							
Q1 (< 2.46)	1820 (19.5)	Reference	0.01	Reference	0.70	Reference	0.03
Q2 (2.46–9.15)	1861 (19.9)	1.00 [093; 1.06]		1.00 [0.93; 1.06]		0.97 [0.90; 1.03]	
Q3 (9.15–15.97)	1898 (20.3)	1.05 [0.98; 1.11]		1.02 [0.96; 1.09]		0.96 [0.89; 1.03]	
Q4 (15.97–24.99)	1923 (20.6)	1.07 [1.00; 1.15]		1.04 [0.98; 1.11]		0.95 [0.87; 1.03]	
Q5 (> 24.99)	1848 (19.8)	1.06 [1.00; 1.14]		1.00 [0.94; 1.07]		0.86 [0.75; 0.97]	

M1: Age as the time scale + energy without alcohol

M2: M1 + (Diabetes, treated hypercholesterolemia, education, family history of hypertension, smoking, physical activity, body mass index)

M3: M2 + Na (mg), K (mg), Mg (mg), AGPIw3 (mg), alcohol (g)

**Table 4:** Hazard ratios of hypertension according to dietary total antioxidant capacity of principal food groups. (*N* = 40,576). E3N Cohort, France 1993–2008

Dietary TAC (mmol/day)		M1		M2		M3	
	N (%) cases	HR [95% CI]	p-trend	HR [95% CI]	p-trend	HR [95% CI]	p-trend
Coffee			0.02		0.03		0.14
Q1 (< 2.46)	1820 (19.47)	Reference		Reference		Reference	
Q2 (2.46–9.15)	1861 (19.90)	0.99 [0.93; 1.06]		1.01 [0.94; 1.08]		0.98 [0.91; 1.05]	
Q3 (9.15–15.97)	1896 (20.28)	1.06 [0.99; 1.14]		1.07 [1.00; 1.14]		1.00 [0.93; 1.08]	
Q4 (15.97–24.99)	1925 (20.59)	1.08 [1.01; 1.16]		1.09 [1.01; 1.16]		0.99 [0.91; 1.09]	
Q5 (> 24.99)	1848 (19.76)	1.07 [0.99; 1.14]		1.07 [0.99; 1.15]		0.91 [0.80; 1.04]	
Tea			0.02		0.12		0.73
Q1 (0)	3814 (40.79)	Reference		Reference		Reference	
Q2 (0.01–0.36)	1412 (15.10)	0.95 [0.89; 1.02]		0.95 [0.89; 1.02]		0.97 [0.91; 1.03]	
Q3 (0.36–1.25)	1411 (15.09)	0.97 [0.91; 1.01]		0.97 [0.91; 1.03]		0.98 [0.92; 1.05]	
Q4 (1.25–2.22)	1385 (14.81)	0.97 [0.91; 1.03]		0.99 [0.92; 1.05]		1.01 [0.94; 1.08]	
Q5 (> 2.22)	1328 (14.20)	0.93 [0.87; 0.99]		0.95 [0.87; 1.01]		0.97 [0.91; 1.04]	
Chocolate			0.18		0. 11		0.23
Q1 (0)	4175 (47.30)	Reference		Reference		Reference	
Q2 (0.02–0.25)	1285 (14.56)	0.98 [0.92; 1.04]		0.96 [0.90; 1.02]		0.99 [0.90; 1.02]	
Q3 (0.26–0.53)	920 (10.42)	0.91 [0.85; 0.98]		0.90 [0.84; 0.97]		0.91 [0.85; 0.98]	
Q4 (0.54–1.30)	1151 (13.04)	0.96 [0.90; 1.03]		0.96 [0.89; 1.02]		0.96 [0.90; 1.03]	
Q5 (> 1.4)	1295 (14.67)	0.95 [0.89; 1.02]		0.94 [0.88; 1.00]		0.95 [0.89; 1.02]	
Fruit and vegetables			0.001		0. 0003		< 0.0001
Q1 (< 0.99)	1907 (20.40)	Reference		Reference		Reference	
Q2 (0.99–1.36)	1836 (19.64)	0.92 [0.86; 0.98]		0.91 [0.85; 0.98]		0.91 [0.85; 0.98]	
Q3 (1.36–1.71)	1869 (19.99)	0.92 [0.86; 0.99]		0.92 [0.86; 0.98]		0.91 [0.85; 0.97]	
Q4 (1.71–2.21)	1815 (19.41)	0.89 [0.83; 0.95]		0.87 [0.82; 0.93]		0.86 [0.80; 0.92]	
Q5 (> 2.21)	1923 (20.57)	0.88 [0.83; 0.95]		0.87 [0.82; 0.94]		0.84 [0.78; 0.91]	
Wine			0.85		0.84		0.008
Q1 (0)	2202 (23.55)	Reference		Reference		Reference	
Q2 (0.01–0.23)	1600 (17.11)	0.97 [0.90; 1.03]		0.96 [0.90; 1.03]		0.95 [0.89; 1.02]	
Q3 (0.23–0.71)	1805 (19.30)	0.95 [0.89; 1.02]		0.95 [0.89; 1.01]		0.92 [0.86; 0.99]	
Q4 (0.71–1.68)	1840 (19.68)	0.93 [0.87; 0.99]		0.93 [0.87; 0.99]		0.88 [0.82; 0.95]	
Q5 (> 1.68)	1903 (20.35)	0.97 [0.91; 1.04]		0.98 [0.92; 1.05]		0.86 [0.77; 0.95]	
Other sources			0.002		0.006		0.01
Q1 (< 0.46)	1994 (21.33)	Reference		Reference		Reference	
Q2 (0.46–0.63)	1913 (20.46)	0.96 [0.89; 1.02]		0.95 [0.89; 1.02]		0.96 [0.90; 1.03]	
Q3 (0.63–0.82)	1848 (19.76)	0.93 [0.87; 0.99]		0.94 [0.88; 1.01]		0.96 [0.89; 1.03]	
Q4 (0.82–1.09)	1748 (18.70)	0.86 [0.80; 0.92]		0.86 [0.80; 0.93]		0.89 [0.82; 0.95]	
Q5 (> 1.09)	1847 (19.75)	0.91 [0.85; 0.99]		0.92 [0.86; 1.00]		0.93 [0.86; 1.01]	

M1: Age as the time scale + energy without alcohol

M2: M1 + (Diabetes, treated hypercholesterolemia, education, family history of hypertension, smoking, physical activity, body mass index)

M3: M2 + Na (mg), K (mg), Mg (mg), AGPIw3 (mg), alcohol (g)

**Fig. 1:**
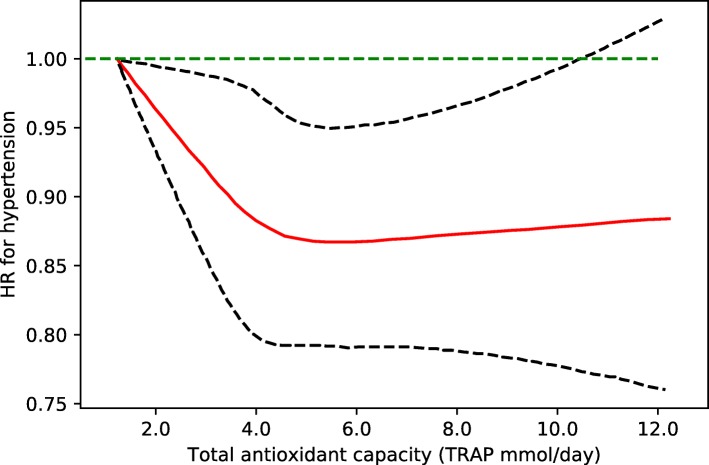
Cubic spline regression model between the total dietary antioxidant capacity (TRAP; mmol/day) and HRs for hypertension; E3N cohort, 1993–2008 (*n* = 40,576). Spline regression: the four knots are the 20th, 40th, 60th and 80th percentiles. The reference value is the minimum total antioxidant capacity. The model was adjusted for smoking status, physical activity, education level, diabetes, hypercholesterolemia, family history of hypertension, energy intake without alcohol, alcohol intake, and BMI. Solid line, HR; dashed lines, 95% CI

The shape of the association between non-coffee dietary TAC and the risk of hypertension is presented in Fig. 1. There was a steep inverse dose-effect relationship between dietary TAC and risk of hypertension up to a TAC value of ca. 5.0 mmoml/day, then a leveling off of the association.

When excluding cases diagnosed during the first 5 years of follow-up (Additional file 1: Table S1; *n* = 38,445), the risk of hypertension associated with non-coffee TAC consumption remained similar (HR_5vs1quintile_: 0.82, 95% CI: 0.75, 0.90; p-trend = 0.0002) while the inverse association with coffee TAC was strengthened (HR_5vs1quintile_: 0.78, 95% CI: 0.67; 0.91; p-trend = 0.01). Associations tended to be slightly weaker when excluding participants with supplemental intake of antioxidants were excluded (Additional file 1: Table S2; *n* = 28,647) (HR_5vs1quintile_: 0.91, 95% CI: 0.83, 1.00; p-trend = 0.06). There was no statistically significant interaction between dietary TAC and BMI, total energy intake, or smoking regarding the risk of hypertension.

## Discussion

In the present study, a high antioxidant capacity was associated with a reduced risk of incident hypertension in a large cohort of French women, especially a high antioxidant capacity from other sources than coffee. Results were stable with time and appeared to be independent of the major risk factors of hypertension, including anthropometry and lifestyle. The spline regression curve demonstrated a steep inverse dose-effect relationship between non-coffee dietary TAC and risk of hypertension, then a leveling off of the association, suggesting that the maximal effect of TAC could be associated with a TAC intake around 5.0 mmol/day.

To the best of our knowledge, this is the first time that an inverse association between the dietary total antioxidant capacity and the risk of hypertension has been shown. Dietary TAC has been previously inversely associated with several outcomes such as stroke [19], myocardial infarction [34], cancer [16, 17], diabetes [20], metabolic disorders [35], and mortality [21]. When hypertensive and normal subjects were compared regarding antioxidant and free radical levels, hypertensive subjects had significantly lower concentrations of HDL-cholesterol, antioxidant enzymes, and ferric reducing antioxidant power but higher total cholesterol and LDL cholesterol concentrations, and higher lipid peroxidation [36].

Previous studies reported inverse associations between intake of antioxidant-rich foods and the risk of hypertension. The DASH diet (Dietary Approaches to Stop Hypertension) [37], based on fruit and vegetables, low-fat dairy products, and whole grains, demonstrated a reduction in blood pressure in both healthy and hypertensive individuals, as well as a reduction in cardiovascular diseases associated with hypertension such as stroke [38]. However, the effect of antioxidant supplementation on blood pressure has not been consistent, especially across genders. The Linxian trial showed a reduced risk of hypertension in the intervention group among men but not among women after 6 years of follow up [39]. In contrast, the SUVIMAX trial did not find any association between antioxidant supplementation and incident hypertension [40]. This suggests that the natural balance between dietary antioxidants could be more efficient for preventing hypertension than specific supplements which may lead to an excess of a given antioxidant and thus to an imbalance of the complex anti-oxidative system.

Oxidative stress results from the excessive production of oxygen free radicals or the decrease of the concentration of antioxidants in the body. It has been suggested that hypertension could indirectly result from a state of imbalance between antioxidants and free radicals [1]. Several mechanisms associated with free radical damage have been suggested, including endothelium dysfunction that would reduce its ability to quench the vasodilator nitric oxide, damage to endothelial cells and to vascular smooth muscle cells, increase in endothelial permeability and intracellular free calcium concentration, collagen deposits leading to the thickening of the vascular media and the narrowing of the vascular lumen, and oxidation of biomolecules such as LDL-cholesterol, a well-known risk factor for atherosclerosis and hypertension [41]. Since antioxidants can stabilize free radicals, they could thereby prevent hypertension by avoiding cell damage.

Dietary TAC represents a global estimate of antioxidants from the diet. However, the ability of diet to increase plasma TAC is a matter of debate. Previous studies reported the ability of various foods including fruit juice [9], wine [10], chocolate [12] onions [11], lettuce [14], or tomato products with extra virgin olive oil [13] to increase plasma TAC. However, a 3-week intervention with fruit juice and vegetable burgers in male smokers failed to modify biomarkers of oxidative stress [15], while another intervention in male smokers of the same duration was shown to increase the plasma oxidative stability, assessed by the oxygen radical absorbance capacity (ORAC) assay [42]. Altogether, intervention studies were of short duration and cannot easily be extrapolated to real-life long term TAC intake.

In our analyses considering the antioxidant capacity of specific food groups, dietary TAC of fruit and vegetables, of wine, and of other miscellaneous foods were inversely associated with hypertension. Fruit and vegetables have been consistently inversely associated with a lower risk of hypertension [43] or coronary heart disease [44], which has been attributed to their antioxidant content. However, high fruit and vegetable intake is a well-known marker of a healthy lifestyle, so that we cannot exclude that the observed association could be a proxy for healthy life choices. However, we also reported an inverse association with the highest quintile of coffee TAC, with TAC from wine, and with TAC for more minor sources. This is more in favor of a true effect of antioxidants on the risk of hypertension.

It is of interest to compare the TAC levels from coffee and those from other sources. While non-coffee TAC was inversely associated with risk from the second quintile on, i.e. from a TAC value above 2.95, an inverse association of a similar magnitude was only observed for coffee TAC from a value of 25, thus nearly ten times higher. This is in line with previously reported questioning about the bioavailability of the various antioxidants from coffee, especially those produced by the Maillard reaction of rather large size [45]. Several studies already reported that coffee TAC was unrelated to the risk of hypertension, possibly due to a balance between favorable antioxidant effects, unfavorable vasoconstriction effects, and possible associations with negative lifestyle factors [46]. Indeed, the effect of coffee on cardiovascular disease is not clear. The most investigated compound is caffeine; studies have shown increased vascular resistance after acute intake of coffee or caffeine, which suggests a vasoconstriction effect [47]. The long-term effect has also been evaluated, and a meta-analysis of randomized controlled trials demonstrated a positive relationship between the number of cups of coffee and changes in systolic pressure [48]. Nonetheless, coffee is also an important source of antioxidants [46]. By partitioning the antioxidant capacity of the diet into coffee and non-coffee components of TAC, we avoided the potential confounding effect of other components of coffee. Our results with almost ten times weaker associations between coffee TAC and hypertension compared to other TAC sources suggest that the antioxidant effect of coffee on hypertension would be largely reduced by potentially lower bioavailability of coffee TAC and adverse cardiovascular effects of caffeine.

Our findings that the association between dietary TAC of chocolate or tea and hypertension was of the same magnitude, but not statistically significant, suggest that in our population with a high proportion of non-consumers of tea or chocolate, consumptions were not high enough to be able to demonstrate any association. A previous study reported no association between tea consumption and hypertension, but an inverse association with chocolate or cocoa consumption [49].

### Strengths and limitations

Strengths of our study are its prospective design since it has been demonstrated that knowledge of hypertension influenced the answer to a dietary questionnaire [50], large sample size, long follow up (15 years) with minimal loss to follow up, large number of cases, and use of a validated diet history questionnaire to evaluate diet and dietary TAC. Our study also has some limitations. Dietary TAC intake was only assessed at baseline thus misclassification of exposure is possible since the dietary habits of participants may change over time. Because of the study design, the measurement error is likely to be non-differential and would tend to attenuate the association. In addition, we did not have dietary TAC values for French foods, and most estimated values related to raw foods. We used the Italian TAC database, but the antioxidant content in foods can be affected by cooking, and by geographic location, climate, and growing conditions of the crop, which may lead to over- or underestimate the TAC content of foods. This should again lead to non-differential misclassification of exposure, and thus reduce the associations, which may therefore be even stronger.

In this study, cases of hypertension were identified through follow-up questionnaires. We assessed the validity of cases of hypertension, and we observed an 82% positive predictive value when we validated self-reported information with the use of a drug reimbursement database. Some degree of misclassification is possible but because hypertension was diagnosed after dietary assessment, it should not be related to the exposure; therefore, this could again potentially attenuate the observed associations. Last, despite the fact that we adjusted the models for all known risk factors for hypertension, residual confounding by some unmeasured or poorly measured factor cannot be totally ruled out.

## Conclusion

Our findings showed that a high TAC diet was associated with a reduced risk of incident hypertension in women, suggesting that promoting a diet naturally rich in antioxidants might help prevent the development of hypertension. These results have to be interpreted cautiously; there are still questions about the absorption, distribution and cellular role still unanswered. Additional studies are needed to further investigate the association of dietary TAC intake with changes in blood pressure levels over time in other settings.

## Additional file

Additional file 1: **Table S1.** Fully adjusted hazard ratios of hypertension according to dietary total antioxidant capacity intake, excluding cases diagnosed in the first 5 years of follow up (*N* = 37,718). E3N Cohort, France 1993–2008. **Table S2.** Fully adjusted hazard ratios of hypertension according to dietary total antioxidant capacity intake, excluding participants with dietary antioxidant supplement intakes (*N* = 28,642). E3N Cohort, France 1993–2008. (DOCX 19 kb)

## Data Availability

Raw data were generated by Inserm at the Institute Gustave Roussy. Derived data supporting the findings of this study are available from the corresponding author upon request.

## Abbreviations

DASH: Dietary Approaches to Stop Hypertension
E3N: The Etude Epidémiologique de femmes de la Mutuelle Générale de l’Education Nationale
HAT reactions: hydrogen atom transfer
HR: Hazard Ratio
METS: Metabolic equivalents
MGEN: Mutuelle Générale de l’Education Nationale
ORAC: oxygen radical absorbance capacity
SET: single electron transfer
TAC: total antioxidant capacity of the diet
TRAP: total radical-trapping ability parameter
