# Supplementary material for: High dietary total antioxidant capacity is associated with a reduced risk of hypertension in French women
Source: Nutr J. 2019 Jun 11;18:31. doi: 10.1186/s12937-019-0456-0 (PMC6560825; doi:10.1186/s12937-019-0456-0)
Supplement: Supplementary file 1 — Table S1. Fully adjusted hazard ratios of hypertension according to dietary total antioxidant capacity intake, excluding cases diagnosed in the first 5 years of follow up (N = 37,718). E3N Cohort, France 1993–2008. Table S2. Fully adjusted hazard ratios of hypertension according to dietary total antioxidant capacity intake, excluding participants with dietary antioxidant supplement intakes (N = 28,642). E3N Cohort, France 1993–2008. (DOCX 19 kb) [file 12937_2019_456_MOESM1_ESM.docx]

**Supplementary table 1. Fully adjusted hazard ratios of hypertension according to dietary total antioxidant capacity intake, excluding cases diagnosed in the first 5 years of follow up (N=37718). E3N Cohort, France 1993-2008.**

| **Dietary TAC (mmol/day)** |  | **M3** |  |
| --- | --- | --- | --- |
|  | **N (%) cases** | **HR [95% CI]** | **p-trend** |
| Non-coffee TAC (minus coffee) |  |  |  |
| Q1 (<2.95) | 1324 (20.39) | Reference | 0.0002 |
| Q2 (2.95 - 3.99) | 1354 (20.86) | 0.96 [0.88; 1.03] |  |
| Q3 (3.99 - 5.52) | 1306 (20.12) | 0.90 [0.83; 0.98] |  |
| Q4 (5.52 – 6.69) | 1273 (19.61) | 0.86 [0.79; 0.93] |  |
| Q5 (>6.96) | 1235 (19.02) | 0.82 [0.75; 0.90] |  |
| Coffee TAC |  |  |  |
| Q1 (<2.46) | 1281 (19.73) | Reference | 0.01 |
| Q2 (2.46 – 9.15) | 1310 (20.18) | 0.93 [0.86; 1.00] |  |
| Q3 (9.15 – 15.97) | 1298 (19.99) | 0.89 [0.82; 0.98] |  |
| Q4 (15.97 – 24.99) | 1321 (20.35) | 0.87 [0.78; 0.97] |  |
| Q5 (> 24.99) | 1282 (19.72) | 0.78 [0.67; 0.91] |  |

M3: Age as the time scale + energy without alcohol + (Diabetes, treated hypercholesterolemia, education, family history of hypertension, smoking, physical activity, body mass index) + Na (mg), K (mg), Mg (mg), AGPIw3 (mg), alcohol (g)

**Supplementary table 2. Fully adjusted hazard ratios of hypertension according to dietary total antioxidant capacity intake, excluding participants with dietary antioxidant supplement intakes (N=28642). E3N Cohort, France 1993-2008.**

| **Dietary TAC (mmol/day)** |  | **M3** |  |
| --- | --- | --- | --- |
|  | **N (%) cases** | **HR [95% CI]** | **p-trend** |
| Non-coffee TAC |  |  |  |
| Q1 (<2.95) | 1309 (20.24) | Reference | 0.06 |
| Q2 (2.95 - 3.99) | 1292 (19.98) | 0.94 [0.87; 1.02] |  |
| Q3 (3.99 - 5.52) | 1273 (19.69) | 0.92 [0.85; 0.99] |  |
| Q4 (5.52 – 6.69) | 1287 (19.90) | 0.90 [0.83; 0.98] |  |
| Q5 (>6.96) | 1305 (20.18) | 0.91 [0.83; 1.00] |  |
| Coffee TAC |  |  |  |
| Q1 (<2.46) | 1280 (19.80) | Reference | 0.40 |
| Q2 (2.46 – 9.15) | 1254 (19.39) | 0.93 [0.86; 1.03] |  |
| Q3 (9.15 – 15.97) | 1285 (19.87) | 0.95 [0.86; 1.03] |  |
| Q4 (15.97 – 24.99) | 1352 (20.91) | 0.97 [0.86; 1.08] |  |
| Q5 (> 24.99) | 1295 (20.03) | 0.91 [0.78; 1.05] |  |

M3: Age as the time scale + energy without alcohol + Diabetes, treated hypercholesterolemia, education, family history of hypertension, smoking, physical activity, body mass index + Na (mg), K (mg), Mg (mg), AGPIw3 (mg), alcohol (g)
